# National Emergency Department Overcrowding Scale as a Predictor for Running an In-Situ Simulation

**DOI:** 10.7759/cureus.108242

**Published:** 2026-05-04

**Authors:** Alexander Croft, Christian Gerhart, Daniel Suarez, Viktoriia Zholob, Nicholas Seidel, Skyler Fustos, Julianne Yeary, Heidi Heitgerd, Laura Frawley, Phillip Asaro, Paul Barach

**Affiliations:** 1 Emergency Medicine, Washington University School of Medicine, St. Louis, USA; 2 Emergency Medicine, Barnes-Jewish Hospital, St. Louis, USA; 3 Anesthesiology, Thomas Jefferson University Hospital, Philadelphia, USA

**Keywords:** clinical operations, emergency medicine, emergency medicine informatics, healthcare simulation, in-situ simulation

## Abstract

Background

In-situ simulation (ISS) is an effective method for training teams and identifying latent safety threats. Operational constraints, such as Emergency Department (ED) overcrowding, can cause simulation cancellation. Cancellation criteria have been proposed; however, there are no objective measures to predict the need to cancel simulations. A reliable cancellation predictor could allow ISS teams to reschedule prior to committing time and resources. The National Emergency Department Overcrowding Scale (NEDOCS) is a ubiquitous index utilized in EDs. We hypothesized that NEDOCS can be an objective and effective predictor of ISS cancellation.

Methods

This is a retrospective observational cohort study examining the relationships between NEDOCS and ISS cancellations at an urban, quaternary ED where overcrowding is common. We compared the mean NEDOCS for days when ISS was canceled (No Go) vs not canceled (Go). A two-tailed t-test was utilized to compare the means between the Go/No Go groups at ISS Start, ISS Start-minus-one hour, ISS Start-minus-two hours, and ISS Start-minus-eight hours.

Results

Mean NEDOCS during the study period was 138.1. The maximum NEDOCS was 286.6 There was a statistically significantly higher mean NEDOCS eight hours prior to ISS start time on days when ISS was canceled (160.6 vs 121.4, p=0.011). At one-hour prior and two-hours prior to start time, there was a non-statistically significant trend towards higher mean NEDOCS on cancellation days. The point-biserial Pearson correlation r value between NEDOCS at T-eight hours and Go/No was -0.61. The point-biserial Pearson correlation r value for all times vs Go/No Go was -0.42 (p=0.0006).

Conclusion

Our data suggests a statistically significant increase in NEDOCS on No Go days compared to Go days for ISS. This association was particularly strong at eight hours prior to start. This suggests that NEDOCS may help identify conditions associated with simulation cancellation the night prior, which may allow for more effective allocation of time and resources.

## Introduction

Many US emergency departments (EDs) suffer from overcrowding and boarding [1,2]. Boarding creates a unique clinical challenge: nurses are tasked with tasks typically associated with an inpatient stay, as well as acute, undifferentiated patients. Simulation has been shown to improve training outcomes and patient outcomes [3]. In-situ simulation (ISS) is a safe and effective method to train and socialize emergency medicine (EM) teams [4,5]. What differentiates ISS from traditional healthcare simulation is the use of the actual clinical equipment, space, and teams that deliver healthcare [6]. Because of the nature of the environment where the scenarios are conducted, ISS can be logistically difficult; however, when compared to traditional healthcare simulation, ISS has the added benefit that it can identify latent safety threats (LSTs). LSTs are defined in healthcare as conditions not readily apparent, that can situationally manifest and threaten patient harm [7]. The advantage to identifying these through a non-patient encounter can mitigate and negate the LST before they can cause a patient harm.

Previous work to address this using ISS has suggested that the use of non-clinical areas, or low volume times in the unit may be of utility [8]. For the simulation team, this alone is a challenge to predict; furthermore, this is compounded by the fact that ISS are typically burdensome on the simulation technician and require advance notice for scenario planning and sustained success. Last-minute cancellations or delays can lead to team frustration, poor time utilization, and ultimately can cause the demise of an ISS program.

While overcrowding is a hospital-wide problem that manifests in the worst way in the ED, measurements have been developed to quantify this problem. The National ED Overcrowding Scale (NEDOCS) score is a previously validated score to quantify ED overcrowding [9]. Because this tool is validated for real-time utilization, it helps hospital administrators understand the needs in the healthcare system and attempt to allocate appropriately [10]. What is unknown is if existing crowding metrics in the ED can be used to forecast the ability to conduct on-shift education or simulation in the ED. The aim of this study is to determine if a set of predefined criteria for ISS completion are associated with the NEDOCS. We hypothesize that higher NEDOCS in the hours prior to an ISS start time will predict the need to cancel a simulation.

This article was previously presented as a meeting abstract at the 25th Annual International Meeting on Simulation in Healthcare, January 10-14, 2025, Orlando, FL.

## Materials and methods

Study description

This is a retrospective observational cohort study of ED overcrowding metrics during the initiation of an in-situ simulation program at a single, high-volume, urban quaternary referral center in St. Louis, Missouri. The ISS program is named the In-Situ Simulation Acute Care (ISSAC) Program.

The ISSAC program is a multidisciplinary, interprofessional group of physicians, nurses, pharmacists, and respiratory therapists, with a goal of improving education, operations, and quality improvement using ISS. From July 2023 to June 2024, this program held over 35 ISS sessions on a range of time-critical topics including trauma, stroke, airway, cardiac conditions, and toxicology.

Location

Barnes-Jewish Hospital is a quaternary care center located in Saint Louis, Missouri. Its ED has over 86,000 annual visits, with over 30% of patients requiring admission. The ED is a Level 1 Trauma center, an American Heart Association (AHA) GOLD Plus Acute myocardial infarction (MI) Award Center, and a Joint Commission Comprehensive Stroke Center. Specific volumes are provided in Table 1.

**Table 1:** Annual statistics of Barnes-Jewish Hospital's emergency department (ED) STEMI: ST-elevation myocardial infarction.

Barnes-Jewish Hospital ED	
Annual Visits	86,067
Admission Rate	31.7%
Level 1 Traumas	1,429
STEMIs	55
Code Stroke Activations	3,739

Timeframe

This study examined the commencement of the ISSAC program at the Barnes-Jewish Hospital. This was between July 2023 and November 2023. The decision to examine this time frame was pre-defined due to the implications of this timeframe for other burgeoning ISS programs.

Go/No Go criteria

To ensure the success of the ISSAC program, a set of contraindications were derived in conjunction with stakeholders from the Washington University School of Medicine Department of Emergency Medicine, Barnes-Jewish Hospital, and nursing leadership. These contraindications are shown in Table 2.

**Table 2:** In-situ simulation contraindications MCI: Mass Casualty Incident.

In-situ simulation contraindications	
Absolute	Relative
“Black” or “Red” Status	Poor provider coverage
Joint commission visits	Nursing staffing shortages
Concurrent MCI drill	-
First responder significant injury in line of duty	-
Severe weather alert	-

Notably, they included a hospital-specific group of color-coded criteria; the determinants for “red” or “black” status are listed in Table 3.

**Table 3:** Determinants of emergency department (ED) status

Determinants of ED status
ED census
Number of intensive-care unit patients
Number of admits without an inpatient bed
Total behavioral health census
Arrivals per hour
Waiting room census
Door-to-room time
Nursing staffing census

NEDOCS score calculation

The NEDOCS was developed by EM physicians in 2004 as a method to universally define ED crowding and continues to be one of the most reliable and highly utilized overcrowding metrics [9,11]. The calculation of NEDOCS includes the following variables: number of ED beds, number of hospital beds, total patients in the ED, patients on ventilators in the ED, number of admits in the ED, waiting time of longest admitted patient and waiting time of longest waiting room patient. A NEDOCS >100 is interpreted as “overcrowded,” and a score of >180 confers “dangerously overcrowded” [9]. Although NEDOCS was not validated at scores above 200 in the original derivation, our ED has found that we frequently have scores higher than 200 and have chosen to display and store the scores without capping at 200. We find that the magnitude of the score, even above 200, seems to have value on face validity. Our instance of Epic (Epic Systems Corporation; Verona, Wisconsin, USA) calculates and stores NEDOCS every 15 minutes. For this study, we retroactively obtained the scores from Epic via data-pull by a study member blinded to the Go/No Go decisions.

Statistics

The mean NEDOCS, for days when ISS was canceled (No Go) vs not canceled (Go) were compared. A two-tailed t-test was utilized to compare the means between the Go/No Go groups at ISS start time, ISS start time-minus-one hour, ISS start time-minus-two hours, and ISS start time-minus-eight hours. Because simulation cancellation was a binary outcome, a point-biserial correlation (reported as Pearson’s r) was used. Data was analyzed in the GraphPad Prism (GraphPad, Boston, Massachusetts, USA) software.

Ethics

This study was approved by The Washington University in St. Louis Institutional Review Board (protocol number 202407095).

## Results

The mean NEDOCS across all days during the study period from July 2023 through November 2023 was 138.1, with a maximum score of 286.6. 

**Figure 1:**
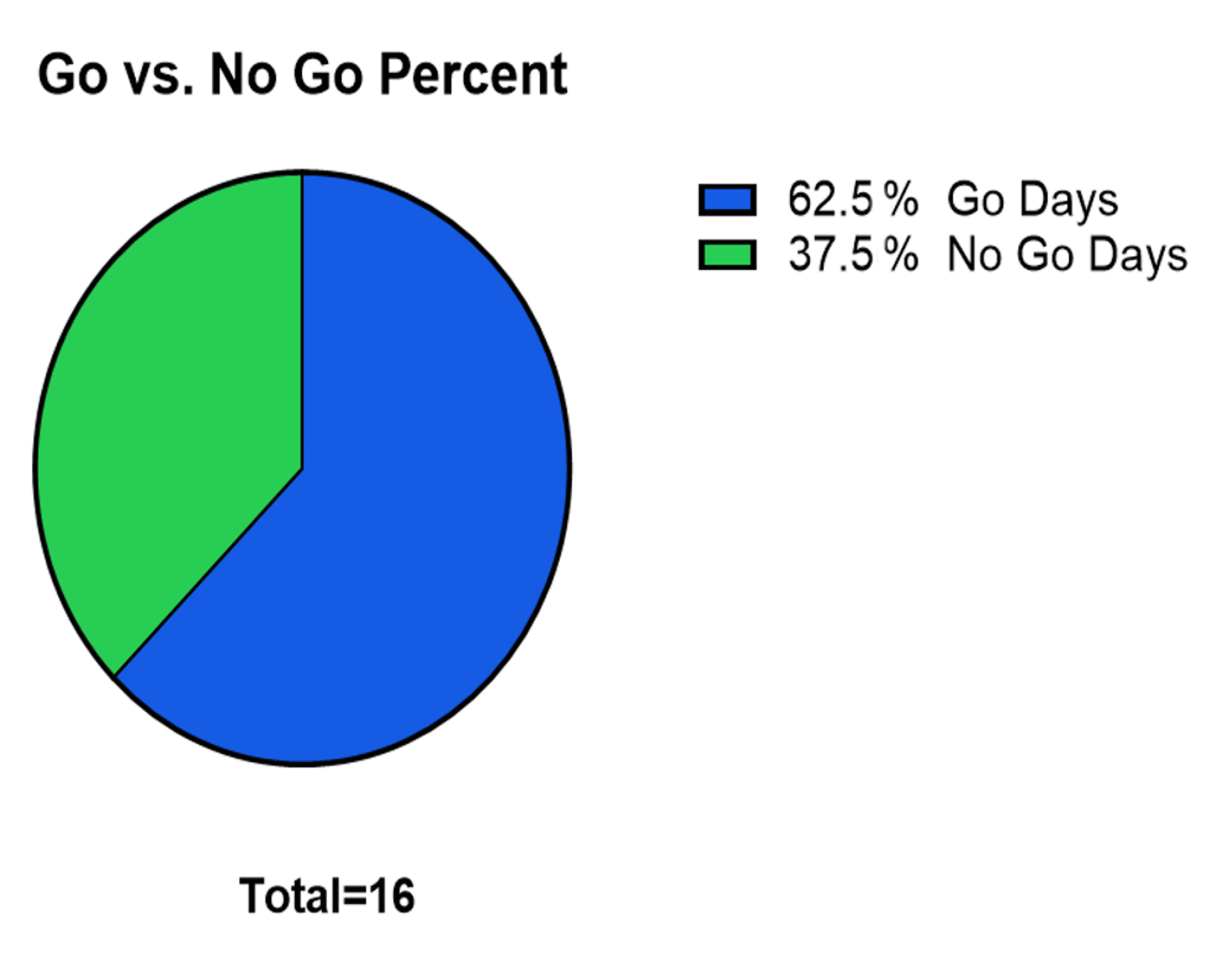
“Go” vs “No Go” dates 'Go' days correspond to days where in-situ simulation sessions were conducted, 'No Go' Days correspond to days where in-situ simulation sessions were unable to be conducted on specific dates within the Barnes-Jewish Hospital Emergency Department. Total number of sessions was 16, cancellation rate was 37.5% (N=6).

From July 2023 to November 2023, 16 ISS sessions were planned. Of these, there were six cancellations, with a cancellation rate of 37.5% (Figure 1). 

**Figure 2:**
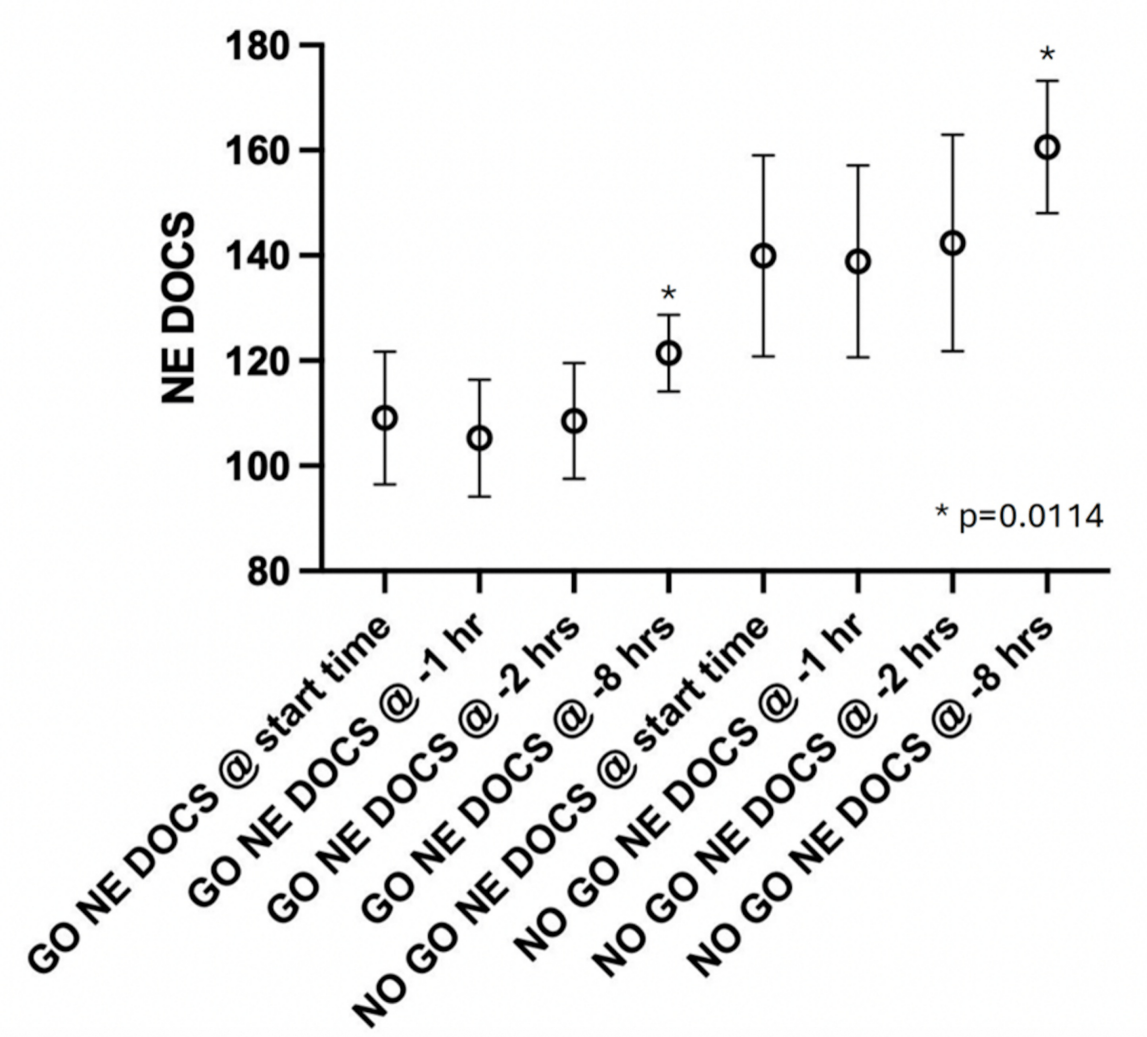
NEDOCS Score for “Go” Versus “No Go” at start of ISS, T-1 hour prior to ISS, T-2 hours prior to ISS, and T-8 hours prior to ISS Error bars represent the standard error of mean (SEM). 'GO NEDOCS' on the x-axis corresponds to dates when in-situ simulation (ISS) sessions were completed, 'NO GO NEDOCS' corresponds to dates when ISS sessions were unable to be completed. NEDOCS: National Emergency Department Overcrowding Scale.

There was a statistically significantly higher mean NEDOCS eight hours prior to ISS start time on days when ISS was canceled compared to not canceled (160.6 vs 121.4, p=0.011) as shown in Figure 2.

At one-hour prior and two-hours prior to start time, we found that there was a non-statistically significant trend towards higher NEDOCS mean scores on days when sessions were canceled. The Pearson r value for the correlation between NEDOCS at minus eight hours and Go/No Go was -0.61. The Pearson r for all times vs Go/No Go was -0.42 (p=0.0006).

## Discussion

During the study period, the simulation cancellation rate was 37.5%. While this is high compared to other published rates [12], it probably reflects the early-stage nature of the ISSAC program and high levels of concern with disruptions in care in the ED.

We found that at eight hours prior to a simulation session, there was a significant measurable difference between the conditions that led to and did not lead to cancellation of the ISS. Interestingly, this difference does not exist two hours or even one hour prior to an ISS. This could be due to a couple of different factors.

First, based on our contraindications for ISS (Table 2), a singular event such as a first responder injured in the line of duty can cause a cancellation, independent of ED overcrowding. This is necessary for the mission of the simulation program but presents a possible confounder in a retrospective nature. However, it is important to note that an event canceled due to an absolute contraindication without high NEDOCS would likely lead to an underestimation of the value of high NEDOCS for cancellations.

Secondly, the sessions performed were done at typically low-volume times, mostly early in the morning. This selection bias unfortunately reflects a necessity when creating an ISS program; to gain provider buy-in and generate positive culture change, it is necessary to not overburden providers in the program’s infancy.

ED overcrowding is not only an EM problem; it is the cumulative result of micro- and macro-level factors in the ED and in the whole hospital system [1]. It is well established that as ED overcrowding and boarding increase, patient outcomes worsen [13]. Additionally, several studies show that ED overcrowding is associated with increased risks of workplace violence, decreased job satisfaction, and clinician burnout [2,14]. These factors must be accounted for when considering additional quality improvement and education training. As our colleagues are increasingly stressed, and the patients get sicker, the “extra” thing will always be the first to go.

ISS has been shown to have many benefits for education, quality improvement, and system operational changes [15-17]. However, the reality of conducting simulation in the clinical environment is that patient care will always come first. Many studies and reviews regarding ISS have cited clinical acuity and volume as a negative determinant of being able to conduct sessions [18]. A decrease in the number of additional educational activities is also compounded by the effect that boarding has had on the education of future trainees; recent studies have shown that boarding has decreased educational benefit for EM trainees [19], with significant impacts on task management. This highlights the need for being able to implement interventions such as ISS into times when the hospital and ED can operationally handle them.

A frustration that is noted by the facilitators of ISS is that the unpredictable nature of the ED leads to cancellation, and often with little notice [8]. This is compounded by the fact that many programs intentionally target times of the day and week when patient volume is lower, such as early morning and weekends. For sustainability of an in-situ program, it is essential to respect the time of the facilitators and the clinical providers to ensure a positive culture of ISS.

The findings of a significant difference between canceled and non-canceled sessions at T-eight highlights a key predictor. Forecasting ED crowding is not a new development, several studies have looked at models that can predict how busy the next shift will be [10,20]. If teams can preemptively predict a cancellation using a readily available overcrowding score, an earlier cancellation allows the facilitator team to better manage their time and could prevent both facilitator and provider frustration. One of the benefits of using NEDOCS is that it is commonly used in most EDs in the United States and is easily calculated for display within the electronic medical-record system. While this study retrospectively examined the feasibility of using a data-driven operational approach of ISS, further prospective studies examining using clinical metrics to operationally forecast would be of great utility and could assist in the development of evidence-based Go/No Go criteria. 

Limitations

The retrospective nature of this study and our relatively small sample size limit the conclusions that can be drawn, but our findings suggest the feasibility of a larger prospective study. Additionally, given that our cancellation criteria included some institution-specific characteristics, there may be limitations in the generalizability to other institutions.

## Conclusions

Simulation training is an essential component of EM training. We found that the NEDOCS scores were higher eight hours prior to cancelled ISS sessions as compared to sessions that we were able to complete. This highlights a potential for forecasting conditions in which exercises could be preemptively cancelled due to ED overcrowding. However, the broader implications of forecasting for ISS program implementation remain poorly understood and warrant further investigation, particularly in improving the feasibility and sustainability of in-situ programs in the ED setting.
